# Nanosponge for Iron Chelation and Efflux: A Ferroptosis‐Inhibiting Approach for Myocardial Infarction Therapy

**DOI:** 10.1002/advs.202305895

**Published:** 2024-04-26

**Authors:** Qingbo Lv, Jun Lin, He Huang, Boxuan Ma, Wujiao Li, Jiawen Chen, Meihui Wang, Xiaoyu Wang, Guosheng Fu, Yun Xiao

**Affiliations:** ^1^ Key Laboratory of Cardiovascular Intervention and Regenerative Medicine of Zhejiang Province Department of Cardiology Sir Run Run Shaw Hospital Zhejiang University School of Medicine Hangzhou 310016 China; ^2^ Department of Cardiovascular Surgery Sun Yat‐sen Memorial Hospital Sun Yat‐sen University Guangzhou 510120 China; ^3^ Qiushi Academy for Advanced Studies Zhejiang University Hangzhou 310058 China

**Keywords:** chitosan, deferoxamine, ferroptosis, myocardial infarction, nanosponge

## Abstract

Myocardial infarction (MI), a consequence of coronary artery occlusion, triggers the degradation of ferritin, resulting in elevated levels of free iron in the heart and thereby inducing ferroptosis. Targeting myocardial ferroptosis through the chelation of excess iron has therapeutic potential for MI treatment. However, iron chelation in post ischemic injury areas using conventional iron‐specific chelators is hindered by ineffective myocardial intracellular chelation, rapid clearance, and high systemic toxicity. A chitosan‐desferrioxamine nanosponge (CDNS) is designed by co‐crosslinking chitosan and deferoxamine through noncovalent gelation to address these challenges. This architecture facilitates direct iron chelation regardless of deferoxamine (DFO) release due to its sponge‐like porous hydrogel structure. Upon cellular internalization, CDNS can effectively chelate cellular iron and facilitate the efflux of captured iron, thereby inhibiting ferroptosis and associated oxidative stress and lipid peroxidation. In MI mouse models, myocardial injection of CDNS promotes sustainable retention and the suppression of ferroptosis in the infarcted heart. This intervention improves cardiac function and alleviates adverse cardiac remodeling post‐MI, leading to decreased oxidative stress and the promotion of angiogenesis due to ferroptosis inhibition by CDNS in the infarcted heart. This study reveals a nanosponge‐based nanomedicine targeting myocardial ferroptosis with efficient iron chelation and efflux, offering a promising MI treatment.

## Graphical abstract

This work addresses the challenges of prevalent iron chelation strategies in myocardial infarction (MI) treatment and proposes a novel therapeutic approach using chitosan–deferoxamine nanosponge (CDNS). This CDNS facilitates efficient iron chelation, promotes iron efflux, and exhibits significant benefits in MI mouse models. This article highlights the potential of this nanomaterial‐driven iron chelation therapy for MI treatment.

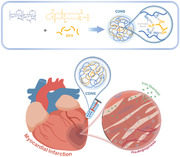

## 1 Introduction

Ischemic heart disease (IHD), particularly myocardial infarction (MI), is the leading cause of mortality globally.^[^ ^1^ ^]^ During cardiac ischemia, the death of cardiomyocytes and their replacement by fibroblasts contribute to irreversible cardiac damage and impaired heart ejection function.^[^ ^2^ ^]^ Imbalanced iron homeostasis plays a significant pathophysiological role in this process. Iron metabolism, which encompasses iron uptake, storage, utilization, and efflux, plays a vital role in cardiac function.^[^ ^3^ ^]^ Excess iron spurs reactive oxygen species (ROS) generation, potentially triggering ferroptosis, an iron‐dependent, lipid peroxidation‐driven form of regulated cell death implicated in cardiovascular diseases including MI.^[^ ^4^ ^]^ Following infarction, ferritin breakdown elevates free iron levels in the affected myocardium,^[^ ^5^ ^]^ intensifying ferroptosis via the Fenton reaction‐mediate conversion of superoxide and hydrogen peroxide to highly reactive hydroxyl radicals.^[^ ^6^ ^]^ This cascade of events inflicts mitochondrial damage and affects calcium homeostasis, exacerbating myocardial injury.^[^ ^7^ ^]^ Chelating excess iron postischemia is thus essential for safeguarding cardiomyocytes from oxidative stress, mitigating injury, and preventing adverse cardiac remodeling.^[^ ^8^ ^]^ Therefore, targeting myocardial ferroptosis through iron chelation is a promising therapeutic approach for improving post‐MI cardiac function.

However, current iron chelation strategies for MI treatment are controversial.^[^ ^9^ ^]^ A major limitation is ineffective intracellular chelation in the myocardium due to the poor cell membrane permeability of hydrophilic chelators, such as the Food and Drug Adminstration (FDA)‐approved deferoxamine (DFO).^[^ ^10^ ^]^ This property impedes their capacity to sequester harmful iron in myocardial tissue. Chelator stability and rapid clearance present further obstacles, as small‐molecule chelators undergo swift hepatic metabolism and have transient circulatory lifespans, diminishing their iron chelation efficacy.^[^ ^11^ ^]^ Additionally, the systemic toxicity of chelators, including hypersensitivity reactions, increased infection risk, and neurotoxicity, raises serious concerns.^[^ ^12^ ^]^ The nonselective activity of the chelators could inadvertently trigger systemic iron depletion, risking deficiencies in body regions requiring iron for normal function. These limitations underscore the need to develop advanced iron chelation strategies that enhance the efficacy, stability, and safety of MI treatment. Importantly, strategies must facilitate iron efflux to enable comprehensive iron removal.^[^ ^13^ ^]^

**Figure 1:**
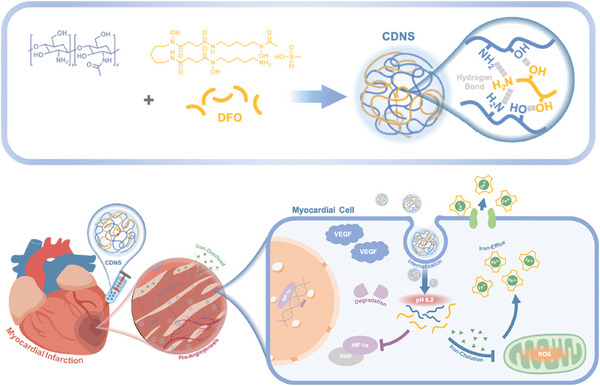
Schematic illustration of CDNS synthesis and its application in the treatment of myocardial infarction. Within the self‐assembled CDNS, CS and DFO are mutually bound through hydrogen bonding, forming a stable structure. CDNS is subsequently injected into the infarcted myocardium of model mice. Upon uptake by cardiomyocytes, CDNS binds to excess free iron within cells and facilitates iron efflux via the membrane protein ferroportin. Moreover, CDNS inhibits HIF‐1α degradation, promotes VEGF transcription, and thereby promotes angiogenesis in ischemic tissues. CDNS: chitosan–deferoxamine nanosponge; CS: chitosan; DFO: deferoxamine; VEGF: vascular endothelial growth factor.

Given these considerations, we propose an iron chelating and efflux nanosponge that ensures efficient endocytosis, prolonged retention, and low systemic toxicity for regulating iron homeostasis in MI. DFO was incorporated into chitosan nanohydrogel scaffolds to fabricate chitosan–deferoxamine nanosponge (CDNS) via coassembly. This engineered nanosponge facilitates direct iron chelation independent of DFO release through its sponge‐like porous hydrogel structure. The abundant hydroxyl, amino, and acetyl groups in CDNS confer specific, efficient iron chelation. With improved biocompatibility, negligible toxicity, and biodegradability, CDNS enables effective cellular uptake. Within myocardial cells, pH‐triggered chitosan (CS) disassembly releases DFO from CDNS, which subsequently forms a complex with iron to facilitate iron efflux. In murine MI models, CDNS administration significantly reduces iron levels in infarcted tissue, relieving post‐MI damage. These benefits arise from the CDNS‐mediated inhibition of ferroptosis, decreased oxidative stress via iron chelation, and stimulation of angiogenesis through HIF‐1α upregulation (**Figure** **1**). Overall, our study revealed a novel nanomaterial‐driven therapeutic strategy for MI treatment, focusing on excess iron removal and angiogenesis promotion in the infarcted heart.

## 2 Results and Discussion

### 2.1 Design, Synthesis, and Characterization of CDNS

**Figure 2:**
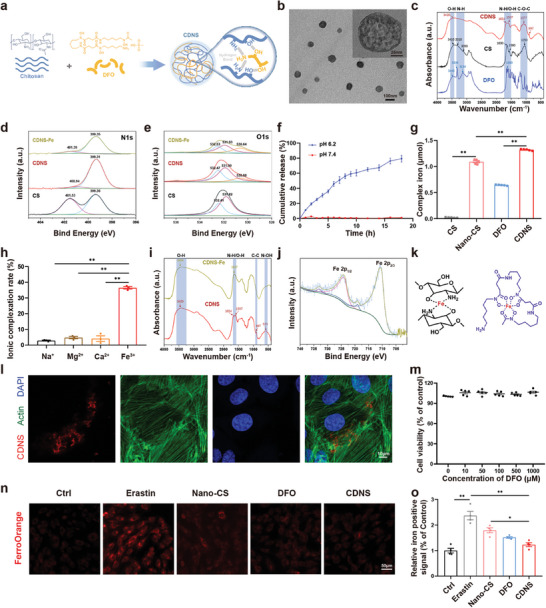
Characterization of the CDNS. a) Schematics of the binding model of CS and DFO. b) Cryo‐transmission electron microscopy (cryo‐TEM) image of CDNS. c) Fourier transform infrared spectroscopy (FT‐IR) spectra of CS, DFO, and CDNS. d,e) X‐ray photoelectron spectroscopy (XPS) analysis of the N 1s and O 1s spectra of CS, CDNS, and CDNS‐Fe. f) DFO drug release behaviors at pH 6.2 and pH 7.4 in vitro. n = 3 per group. g) Fe^3+^ chelation efficiency of free chitosan, Nano‐CS, DFO, and CDNS. (DFO at a concentration of 100 µg mL^−1^). n = 5 per group. h) Efficiency of CDNS in chelating different metallic ions (Na^+^, Mg^2+^, Ca^2+^, and Fe^3+^). n = 3 per group. i) FT‐IR spectra of CDNS and CDNS‐Fe. j) XPS analysis of the Fe 2p spectrum of the CDNS‐Fe complex. k) The possible chemical bonds between CDNS and iron ions. l) Representative images of fluorescence staining of H9c2 cells treated with FITC‐labeled CDNS for 30 min. Scale bar: 10 µm. m) Viability of H9c2 cells after incubation with various concentrations of CDNS. N = 5 per group. n,o) Representative images and quantification of free iron concentrations in H9c2 cells subjected to FerroOrange staining. n = 4 cells per group. Scale bar: 50 µm. ^*^ *p* < 0.05. ^**^ *p* < 0.01.

Both CS and DFO possess a multitude of hydroxyl and amino groups,^[^ ^14^ ^]^ enabling robust structures to form through hydrogen bonding (**Figure** **2**a). The fabrication process of CDNS, as outlined in Figure S1a (Supporting Information), involved the self‐assembly of CS (1.25 mg mL^−1^) and encapsulation of DFO (1 mg mL^−1^) at pH 7.4. The morphology and dimensions of the CDNS were evaluated by transmission electron microscopy (TEM) (Figure S1b, Supporting Information) and scanning electron microscopy (SEM) (Figure S1c, Supporting Information), which revealed uniformly distributed nanoparticles with a rough surface and an average size of ≈50 nm. Dynamic light scattering (DLS) findings (Figure S1c, Supporting Information, insert) corroborated these results. Notably, cryo‐transmission electron microscopy (cryo‐TEM) further revealed a distinctive contrast within the nanoparticles, substantiating the presence of a sponge‐like nanosponge structure within the CDNS (Figure 2b). Elemental mapping through energy‐dispersive spectroscopy (EDS) (Figure S1d,e, Supporting Information) and X‐ray photoelectron spectroscopy (XPS) (Figure S1f, Supporting Information) verified the C, N, and O compositions aligning with CS ((C_6_H_11_NO_4_)_n_) and DFO (C_25_H_48_N_6_O_8_). Moreover, the zeta potential of CDNS exhibited a negative surface charge of ≈−4.40 ± 0.24 mV, implying interplay between the positively charged CS and DFO (Figure S1g, Supporting Information).

Fourier transform infrared spectroscopy (FT‐IR) was used to analyze the interactions between CS and DFO (Figure 2c). CS displayed prominent ─OH stretching at 3410 cm^−1^ and NH_2_ peaks at 3310 and 3099 cm^−1^. DFO exhibited moderate ─OH stretching at 3445 cm^−1^ and N─H stretching at 3336 cm^−1^. Upon crosslinking DFO and CS, the ─OH vibration shifted from 3445 to 3420 cm^−1^ as the amino peak broadened, which is indicative of hydrogen bond formation through hydroxy/amino groups. Significant N─H bending and N─O asymmetric stretching deviations at 1654 and 1597 cm^−1^ implied amino group‐mediated hydrogen bonding. Moreover, the absorption of CS at 1050 cm^−1^, which is typical of asymmetric vibrations of glycosidic bonds (C─O─C), decreased to 1077 cm^−1^ in the CDNS spectrum due to spatial structural changes triggered by the hydrogen bonding between CS and DFO. These significant absorbance shifts were unequivocally attributable to the hydrogen bonding occurring between CS and DFO during CDNS formation, which served as the principal mechanism for maintaining nanostructure stability. XPS of N 1s, O 1s, and C 1s (Figure 2d,e; Figure S1h, Supporting Information) further validated the occurrence of CS‐DFO interactions. The N 1s spectra of CS displayed characteristic peaks at 399.36 and 401.53 eV, corresponding to NH and NH_2_, respectively. In CDNS, the peak of NH_2_ was significantly diminished, indicating nitrogen‐mediated CS‐DFO binding. Similarly, the CS O 1s peaks at 532.41 and 531.82 eV shifted to 532.47 and 531.9 eV, respectively, in the CDNS spectrum, with a new peak at 530.66 eV (C─O─C, ─OH, and C═O). This result reveals oxygen‐based hydrogen bonding. FT‐IR and XPS analyses revealed crucial molecular interactions between CS and DFO via hydrogen bonding, which maintained CDNS stability.

We evaluated the quantity of free DFO released from the CDNS system via high‐performance liquid chromatography (HPLC) to determine whether crosslinked DFO could be discharged from CDNS.^[^ ^15^ ^]^ Figure S2 (Supporting Information) shows the standard curve of released DFO detected using HPLC. Remarkably, at pH 6.2, the CDNS system released ≈80% of the DFO, whereas minimal DFO release was observed at pH 7.4, indicating its impressive pH‐responsive release characteristics (Figure 2f). This pH sensitivity is particularly relevant in the context of ischemic tissues, which often exhibit a slightly lower pH due to hypoxia and subsequent lactate accumulation^[^ ^16^ ^]^; thus, the CDNS may offer targeted therapeutic benefits under these pathological conditions. Taken together, these results suggested the successful synthesis of CDNS with commendable pH‐dependent release properties.

### 2.2 CDNS Possessed Iron‐Chelating Ability

A quantitative analysis of the binding capacity was performed to verify the iron chelation ability of CDNS. DFO and iron ions form a colored Fe‐DFO complex for iron quantification (Figure S3a,b, Supporting Information). The chitosan solution alone failed to chelate iron, while the chitosan nanosponge (Nano‐CS) without DFO substantially improved iron chelation, highlighting the key role of chitosan crosslinking in iron sequestration. Moreover, the iron adsorption capacity of CDNS significantly exceeded that of both free DFO and Nano‐CS, suggesting a synergistic interaction between DFO and chitosan (Figure 2g). Intriguingly, CDNS exhibited selective iron chelation, with markedly lower adsorption of sodium, magnesium, and calcium than of iron (Figure 2h). Concurrently, we observed that the standalone CS and DFO exhibited minimal affinity for these ions (Na^+^, Mg^2+^, and Ca^2+^), as shown in Figure S4 (Supporting Information). This selectivity signifies the preferential iron chelation of CDNS, which is beneficial for therapeutic efficacy while minimizing side effects.

The FT‐IR analysis revealed the chelation mechanism (Figure 2i). In the CDNS‐Fe complex, N─H bending at 1654 cm^−1^ and N─O asymmetric stretching at 1597 cm^−1^ converged into a strong 1637 cm^−1^ peak, suggesting O‐ and N‐mediated iron binding from eliminated N─O vibrations and a shifted amino peak. Meanwhile, C─C stretching at 897 cm^−1^ disappeared as iron binding altered the C─C chain. The minor 569.96 cm^−1^ N─OH bending vibration peak also vanished, likely from iron displacing OH hydrogens. The N1s (Figure 2d), O1s (Figure 2e), and C 1s (Figure S1h, Supporting Information) XPS spectra of the CDNS‐Fe complex further showed that iron‐bound through oxygen and nitrogen, which was consistent with the FT‐IR results. This finding was further confirmed by the Fe 2p_3/2_ and Fe 2p_1/2_ binding energies, which were 710.37 and 723.88 eV, respectively, indicating the integration of iron into the CDNS matrix (Figure 2j). Together, the FT‐IR and XPS results verified the iron chelation capacity of CDNS via its oxygen/nitrogen groups, highlighting its potential for ferroptosis therapy (Figure 2k).

Following successful synthesis and chemical characterization, we evaluated CDNS biocompatibility and uptake in H9c2 cardiomyocytes. Effective cellular uptake of CDNS was observed after a 30 min incubation period with H9c2 cells, as indicated by the intracellular presence of red CDNS signals (Figure 2l). TEM also revealed the presence of nanoparticles within the endocytic vesicles (Figure S5a,b, Supporting Information). A cell counting kit‐8 (CCK‐8) assay revealed no significant CDNS cytotoxicity at concentrations up to 1000 µm (Figure 2m), suggesting promising cardiomyocyte compatibility. These results highlight the promising compatibility of CDNS with cardiomyocytes. Subsequently, the intracellular iron chelation capability of CDNS was investigated using an erastin‐induced ferroptosis cell model. Ferroptotic cells were subjected to different treatments and subsequently stained with FerroOrange to label cellular iron for a fluorescence microscopy analysis. As anticipated, untreated ferroptotic cells displayed high iron levels. However, compared with ferroptotic cells, cells treated with Nano‐CS, DFO, or CDNS exhibited reduced levels of intracellular iron. Due to the synergistic effects of Nano‐CS and DFO, the reduction effect of CDNS was most pronounced, confirming the efficacy of CDNS in sequestering cellular iron (Figure 2n,o).

### 2.3 CDNS Inhibited Ferroptosis and Attenuated the Oxidative Stress of Cardiomyocytes In Vitro

**Figure 3:**
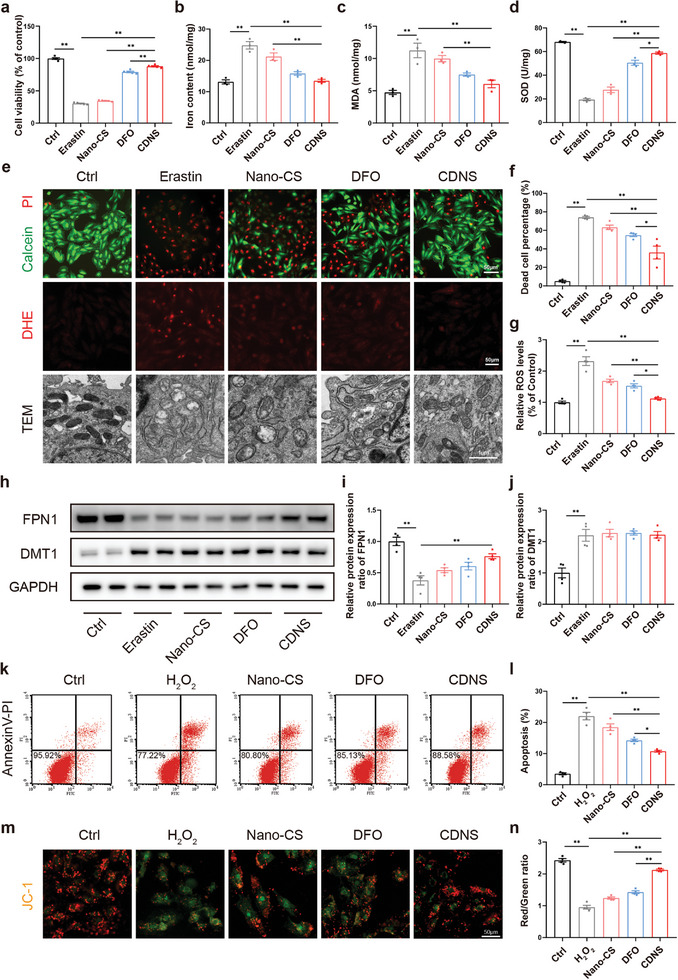
CDNS inhibited ferroptosis and protected against oxidative damage in H9c2 cells. a) CCK‐8 assay for the evaluation of the relative viability of H9c2 cells treated with 10 µm erastin for 24 h. n = 5 per group. b) The iron content after different treatments was evaluated with an iron colorimetric assay kit. n = 4 per group. c,d) Relative MDA levels and SOD activity after different treatments. n = 4 per group. e) Representative images of calcein AM/PI staining, DHE staining, and TEM after erastin stimulation and different treatments. f) Quantification of dead/live H9c2 cells subjected to calcein AM/PI staining. n = 4 cells per group. g) Quantification of DHE staining after erastin stimulation and different treatments. n = 4 cells per group. h–j) Representative immunoblot image and quantification of the relative protein expression of FPN1 and DMT1 in H9c2 cells. n = 4 per group. k,l) Flow cytometry analyses of apoptosis after stimulation with hydrogen peroxide and different treatments evaluated by Annexin V‐PI staining. n = 4 per group. m,n) Mitochondrial membrane potential of H9c2 cells after stimulation with hydrogen peroxide and different treatments evaluated by JC‐1 staining. Relative ratios of red versus green fluorescence intensities in H9c2 cells subjected to JC‐1 staining. Scale bar: 50 µm. n = 4 per group. ^*^ *p *< 0.05. ^**^ *p *< 0.01.

Following the confirmation of the iron chelation capability of CDNS, we investigated its ability to inhibit ferroptosis in vitro. The CCK‐8 assay was utilized to evaluate the cytoprotective effect of CDNS on erastin‐induced H9c2 ferroptosis. **Figure** **3**a shows that compared with the control treatment, the CDNS treatment improved viability by 289%, reaching 87.9% of the normal cell viability. This result highlights the protective effects of CDNS. The accumulation of free iron in H9c2 cells was quantified using an iron colorimetric assay kit. Figure 3b shows that the erastin‐induced group exhibited a significant increase in the accumulation of free iron in ferroptotic cells. In contrast, both DFO and CDNS decreased the free iron content, illustrating the efficient chelation of intracellular free iron. The significant increase in cell viability and mitigation of iron accumulation highlighted the potential of CDNS in ferroptosis‐related therapeutic strategies.

The deposition of free iron subsequently triggers lipid peroxidation, which leads to increased malondialdehyde (MDA) levels and decreased superoxide dismutase (SOD) levels.^[^ ^17^ ^]^ Intriguingly, CDNS inhibited MDA production and stimulated SOD production (Figure 3c,d), indicating that CDNS could attenuate lipid peroxidation. Calcein/PI staining further probed the effects on erastin‐induced H9c2 ferroptosis (Figure 3e). The percentages of calcein‐positive (live) cells were significantly greater in the CDNS group than in the DFO group (Figure 3f). Lipid peroxidation generates ROS, key ferroptosis inducers.^[^ ^4b^ ^]^ DHE staining validated the antioxidant effects of CDNS on erastin‐induced oxidative stress (Figure 3g). Additionally, TEM revealed that erastin exposure caused pronounced morphological changes in mitochondria, including an increased membrane density and loss of cristae (Figure 3e). Compared with erastin treatment, CDNS treatment effectively counteracted these mitochondrial disruptions, as evidenced by a reduction in the mitochondrial membrane density, restoration of the cristae architecture, and an enhancement of outer membrane integrity. Overall, through its iron‐chelating activity, CDNS confers protection against ferroptotic alterations, preserves mitochondrial function, and curtails oxidative stress‐induced damage.

We examined the iron transporters ferroportin 1 (FPN1, efflux) and divalent metal transporter 1 (DMT1, import) by western blotting to determine whether CDNS promoted iron transport.^[^ ^5^ ^]^ In ferroptotic cells, we observed a significant downregulation of FPN1 protein expression and a marked upregulation of DMT1 expression, which are factors that lead to intracellular iron overload. In contrast, compared with ferroptotic cells, CDNS markedly upregulated FPN1 without decreasing DMT1, implying an increased capacity for intracellular iron clearance (Figure 3h–j). Notably, the regulatory capacity of the CDNS group surpassed that of the DFO group, due primarily to the lower cellular uptake rate and intracellular concentration of DFO. Overall, these findings indicated that CDNS provided substantial protection against ferroptosis and oxidative damage in cardiomyocytes by chelating iron and promoting its removal.

By recognizing excessive oxidative damage as a key contributor to cardiomyocyte injury,^[^ ^18^ ^]^ we simulated oxidative stress injury with hydrogen peroxide (H_2_O_2_) to evaluate the resistance conferred by CDNS. Lactate dehydrogenase (LDH) assays showed that Nano‐CS, DFO, and CDNS all mitigated H_2_O_2_‐induced death, with CDNS exhibiting the most significant protective effect (Figure S6a, Supporting Information). Annexin V‐PI staining confirmed the superior protective effect of the CDNS (Figure 3k,l). Furthermore, DHE staining revealed that H_2_O_2_ substantially increased ROS levels, while compared with Nano‐CS and DFO, CDNS provided more substantial protection against H_2_O_2_‐induced oxidative damage (Figure S6b,c, Supporting Information). As oxidative stress often triggers mitochondrial dysfunction, leading to cell injury or death,^[^ ^19^ ^]^ tetraethylbenzimidazolylcarbocyanine iodide (JC‐1) staining was used to assess the effect of CDNS on H_2_O_2_‐induced changes in the mitochondrial membrane potential.^[^ ^20^ ^]^ CDNS significantly increased the ratio of red (healthy mitochondria) to green fluorescence (mitochondria with an impaired membrane potential) in H_2_O_2_‐treated H9c2 cells, effectively counteracting mitochondrial damage (Figure 3m,n). These findings illustrated a potential role for CDNS in preserving mitochondrial health under oxidative stress. Finally, a tube formation assay in human umbilical vein endothelial cells (HUVECs) was performed to evaluate the angiogenic potential of CDNS in vitro. We found that the angiogenic ability of the CDNS group was significantly greater than that of the control group (Figure S7a,d, Supporting Information). This increase is attributed to the ability of CDNS to impede ferroptosis and mitigate oxidative stress, thereby augmenting endothelial cell viability and angiogenic function.

### 2.4 CDNS Enabled Sustainable Heart Retention

**Figure 4:**
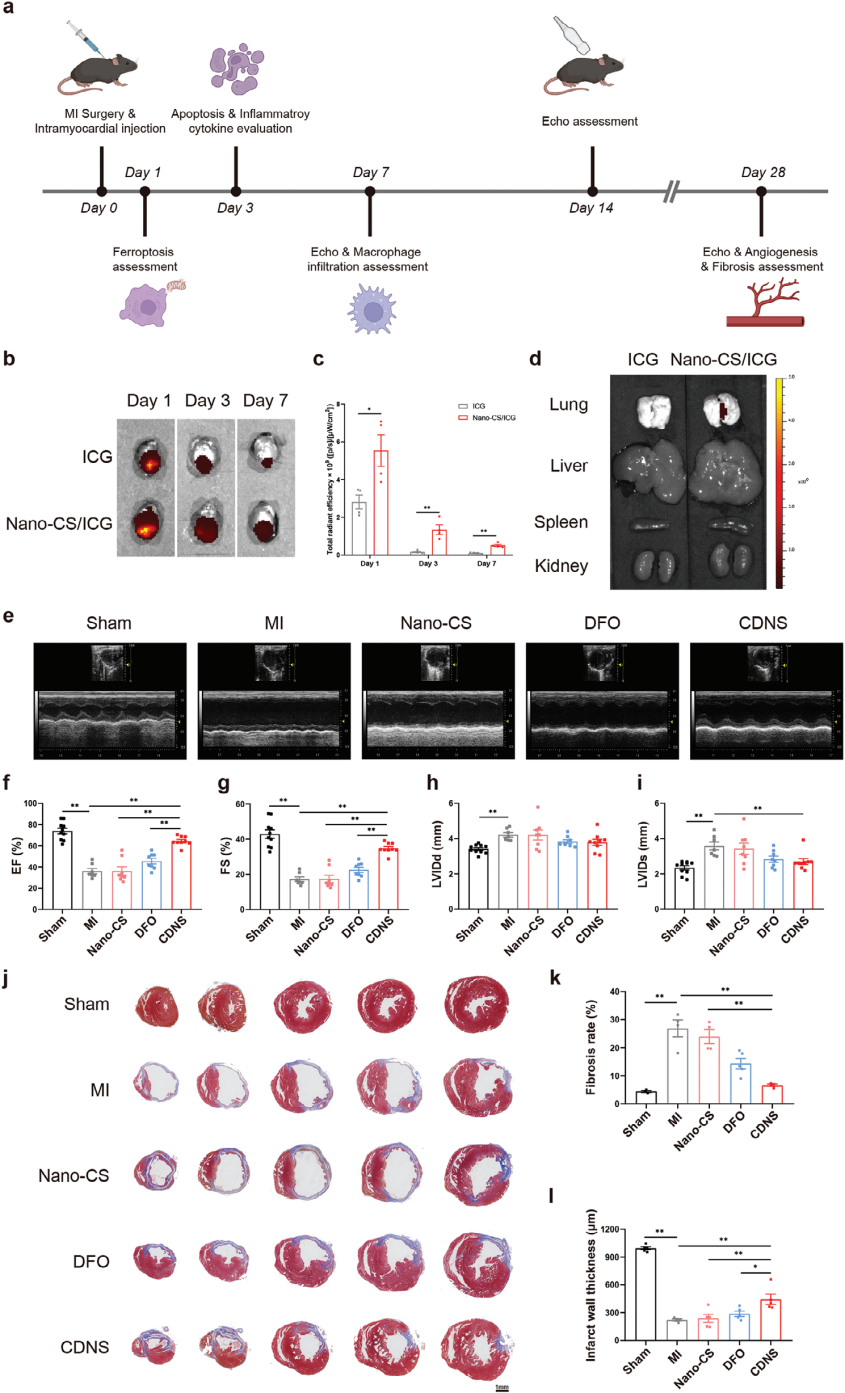
CDNS preserved cardiac function and decreased the infarcted area of the heart after MI. a) Schematic illustration of the in vivo experiment. b) Representative ex vivo fluorescence images of the hearts of MI model mice at 1, 3, and 7 days after the injection of free ICG or ICG‐incorporated Nano‐CS. c) Quantitative analysis of the fluorescence intensities of MI hearts after the injection of free ICG or ICG‐incorporated Nano‐CS. n = 4 for each group. d) Representative *ex vivo* fluorescence images of dissected organs on day 7 after treatment. e) Representative echocardiography images of the hearts of different groups at 28 days after MI. f–i) Quantification of the EF, FS, LVIDd, and LVIDs of the hearts of different groups at 28 days after MI. n = 10 for the sham group, n = 7 for the MI group, n = 8 for the Nano‐CS group, n = 8 for the DFO group, and n = 9 for the CDNS group. j–l) Representative images of Masson's trichrome staining and quantification of fibrotic areas and the infarct wall thickness of the hearts in different groups at 28 days after MI. Scale bar: 1 mm. Quantification of fibrotic areas: n = 3 for the sham group, n = 4 for the MI group, n = 4 for the Nano‐CS group, n = 5 for the DFO group, and n = 4 for the CDNS group. Quantification of the infarct wall thickness: n = 5 animals per group. ^*^ *P* < 0.05. ^**^ *p* < 0.01.

Upon clarifying the inhibitory effect of CDNS on ferroptosis in cardiomyocytes, we investigated the in vivo therapeutic effect of CDNS on a murine MI model (**Figure** **4**a). DFO is rapidly metabolized by the liver and kidneys, which significantly shortens its circulatory half‐life and limits its efficacy in the myocardium.^[^ ^21^ ^]^ We focused on whether the integration of DFO into chitosan hydrogels could overcome its rapid clearance and enhance myocardial retention. We labeled the chitosan nanosponge with the water‐soluble fluorescent dye indocyanine green (ICG),^[^ ^22^ ^]^ which shares solubility properties with DFO. Post‐MI, we administered free ICG or ICG‐loaded Nano‐CS (Nano‐CS/ICG) into the myocardium and traced the fluorescent signals at 1, 3, and 7 days postinjection using *ex vivo* imaging. Hearts injected with Nano‐CS/ICG displayed more robust fluorescence signals than those injected with free ICG, with the signal persisting in the heart of Nano‐CS/ICG‐treated mice for at least 7 days post‐MI (Figure 4b,c). Furthermore, no discernible fluorescence appeared in the liver, spleen, or kidneys of either group and was localized to the heart and lungs (Figure 4d). This result implies that CDNS enables sustained drug release specifically in the heart with minimal off‐target effects, overcoming the brief circulatory half‐life of DFO. The incorporation of DFO into chitosan hydrogels highlights the potential to expand the therapeutic applications of this iron chelator nanosponge. The specificity of CDNS delivery with minimal off‐target organ distribution significantly reduces systemic side effects. These promising results indicate that chitosan nanosponge hydrogels may serve as a delivery platform to enhance MI management through improved pharmacokinetics and localized treatment.

### 2.5 CDNS Improved Cardiac Function and Preserved the Heart Structure Post‐MI

Encouraged by the outstanding retention properties of the chitosan nanosponge system in the heart, we applied CDNS to the MI model to observe its therapeutic effects. The model was established by ligating the left anterior descending artery in mouse hearts, with successful induction confirmed by typical electrocardiographic changes, including QRS complex widening and a reduction in the R‐S wave amplitude (Figure S8, Supporting Information). Serum biomarkers of cardiac injury, cardiac troponin I (cTnI), cardiac troponin T (cTnT), and creatine kinase‐MB (CK‐MB), were measured at 24 h post‐MI, revealing notable elevations indicative of myocardial damage (Figure S9a–c, Supporting Information). In comparison, CDNS‐treated mice exhibited substantially lower levels of these enzymes, indicating a reduction in infarct severity.

Subsequently, we examined whether CDNS could improve cardiac function and structure in vivo. Cardiac ultrasonography was conducted on days 7, 14, and 28 post‐MI. Representative long‐axis echocardiography images from each group at 7, 14, and 28 days post‐MI are shown in Figure S10a (Supporting Information) and Figure 4e. As depicted in Figure S10b–e (Supporting Information), MI surgery substantially impaired cardiac function, as evidenced by a markedly decreased ejection fraction (EF) (70.58 to 36.55%) and fractional shortening (FS) (39.56 to 17.53%) in the MI group compared with the sham group. Concurrent structural remodeling was observed, as evidenced by an increased left ventricular internal dimension during diastole (LVIDd) (3.412 to 4.218 mm) and systole (LVIDs) (2.344 to 3.585 mm). Notably, both DFO and CDNS improved the EF and FS at 7 days post‐MI. Furthermore, CDNS provided a notably greater enhancement in EF and FS than DFO at 14 days (Figure S10f,g, Supporting Information). This improvement was even more pronounced at 28 days, with CDNS inducing a substantial 28.44% increase in EF (Figure 4f–i). While LVIDd was unchanged (Figure S10d,h, Supporting Information; Figure 4h), CDNS beneficially reduced LVIDs from day 7 to 28 post‐MI, with a reduction of 0.851 mm compared with that of the MI controls on day 28 (Figure S10e,i, Supporting Information; Figure 4i). Following the procurement of the infarcted hearts at 28 days post‐MI, morphometric analyses were performed after Masson's trichrome staining. As depicted in Figure 4j–l, the CDNS group displayed a reduced fibrotic area and increased infarct wall thickness compared to the other groups, suggesting less left ventricular damage. The substantial CDNS‐mediated improvements in EF/FS over DFO highlight its potential for superior efficacy in post‐MI recovery. The superior retention properties of CDNS accounted for this enhanced efficacy, increasing the therapeutic compound's dwell time within cardiac tissue and thereby enabling more effective tissue repair. The results also suggested that CDNS had a positive impact on the cardiac structure. Specifically, the reduction in the fibrotic area and the increased infarct wall thickness implied a lesser degree of cardiac remodeling, which was associated with a poor prognosis for MI patients.

### 2.6 CDNS Inhibited Ferroptosis and Apoptosis In Vivo

**Figure 5:**
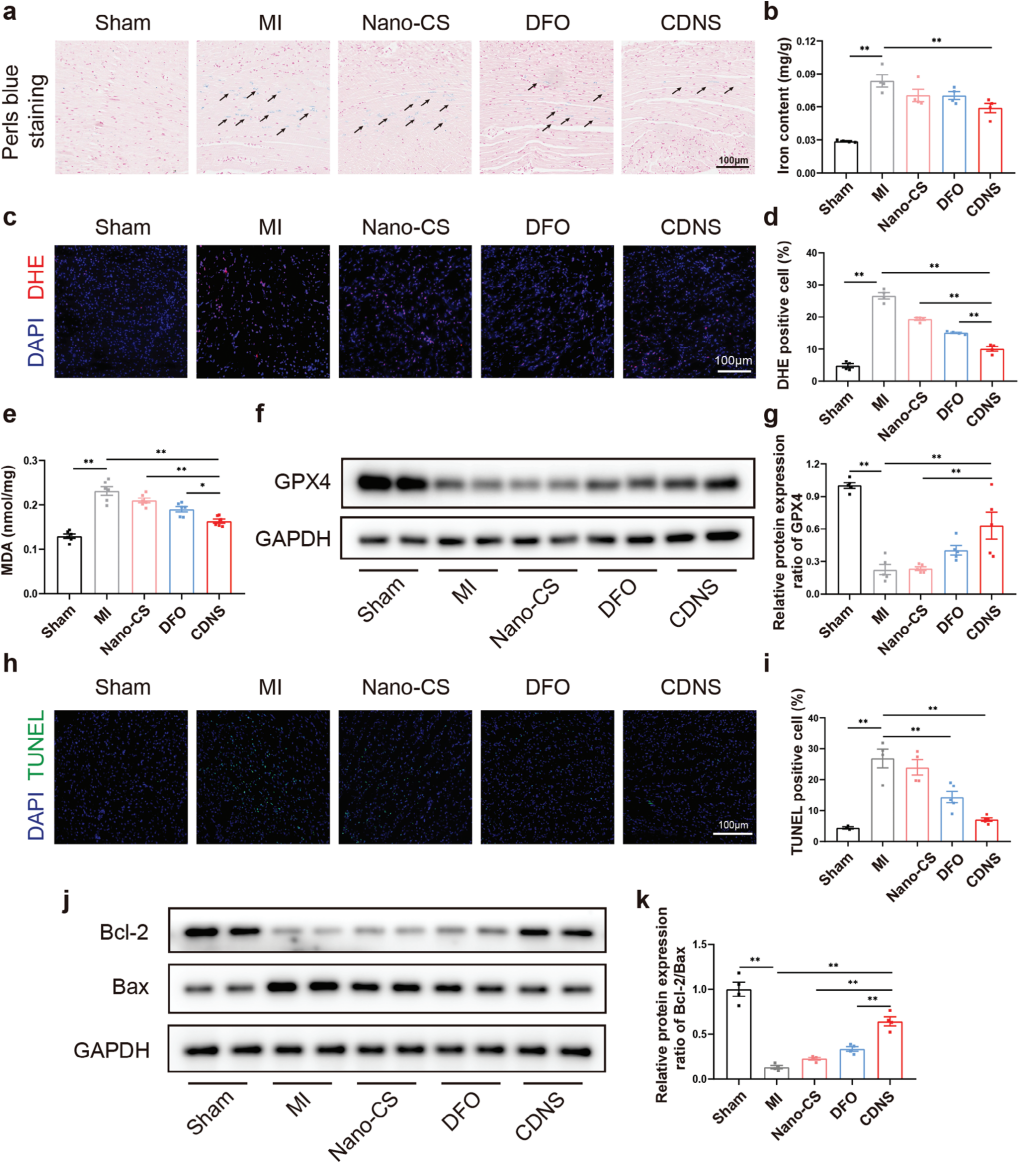
CDNS inhibited ferroptosis and apoptosis in the heart tissues of MI mice. a) Representative images of MI mouse heart tissue sections subjected to DAB‐enhanced Perls blue staining on day 1 after MI. Scale bar: 100 µm. b) Quantification of the iron content by an iron colorimetric assay kit on day 1 after MI. n = 4 per group. c,d) Representative images and quantification of DHE staining on day 1 after MI. Scale bar: 100 µm. n = 4 per group. e) Quantification of MDA levels on day 1 after MI. n = 4 per group. f,g) Representative immunoblot image and quantification of the relative protein expression of GPX4 in the heart on day 1 after MI. n = 4 per group. h,i) Representative image and quantification of TUNEL‐positive cells in the infarcted heart at 3 days after MI. Scale bar: 100 µm. n = 4 per group. j,k) Representative immunoblot image and quantification of the relative protein expression of Bax and Bcl‐2 in the heart at 3 days after MI. n = 4 per group. ^*^ *p* < 0.05. ^**^ *p* < 0.01.

The remarkable cardiac functional/structural improvements and reduced remodeling prompted an investigation into the underlying therapeutic mechanisms. Building on the established link between ferroptosis and MI pathogenesis,^[^ ^4^, ^23^ ^]^ we examined the inhibitory effects of CDNS on myocardial ferroptosis post‐MI in vivo. Iron deposition and contents were significantly increased in the MI group compared with the sham group (**Figure** **5**a,b). Notably, compared with the MI group, CDNS potently suppressed iron deposition. DHE staining was used to assess oxidative stress. MI increased the levels of ROS in the infarcted heart, while CDNS most substantially reduced the number of DHE‐positive cells, indicating antioxidant effects (Figure 5c,d). MI also markedly elevated the levels of the lipid peroxidation marker MDA, which CDNS suppressed (Figure 5e). MI further decreased the expression of the key antioxidant enzyme glutathione peroxidase 4 (GPX4), which protects against lipid peroxidation and ferroptosis by converting phospholipid hydroperoxides into alcohol.^[^ ^24^ ^]^ This finding indicated weakened anti‐ferroptotic defenses and increased levels of toxic lipid ROS. CDNS markedly increased GPX4 expression (Figure 5f,g). The significant GPX4‐mediated effects highlight the potential of CDNS to enhance defenses against detrimental ferroptosis and oxidative stress in myocardial preservation post‐MI.

Emerging evidence underscores the complex interplay between apoptotic and ferroptotic pathways, suggesting that signal interactions may influence mutual outcomes.^[^ ^25^ ^]^ Specifically, the amplification of ROS generated by ferroptosis further catalyzes the process of cellular apoptosis.^[^ ^26^ ^]^ Thus, mitigating both pathways is likely imperative for comprehensive cardioprotection post‐MI. In pursuit of a thorough understanding of the suppressive effect of CDNS on programmed cell death, we assessed the apoptotic state in the context of MI at 3 days post‐MI. This experiment involved conducting terminal deoxynucleotidyl transferase dUTP nick end labeling (TUNEL) assays to determine the potential antiapoptotic impact of CDNS at this juncture. Our findings indicated that compared with the MI group, CDNS significantly reduced the number of TUNEL‐positive cells, suggesting that CDNS had antiapoptotic effects (Figure 5h,i), although no difference was detected between CDNS and DFO. Furthermore, the reduced Bcl‐2/Bax ratio in the MI controls indicated apoptosis, while the CDNS‐treated groups displayed elevated ratios, validating the anti‐apoptotic effects (Figure 5j,k). Taken together, these results suggest that CDNS potently inhibits ferroptosis and apoptosis to preserve myocardial tissue post‐MI, explaining the observed functional/structural improvements.

### 2.7 CDNS Alleviated Inflammation and Promoted Angiogenesis After MI

**Figure 6:**
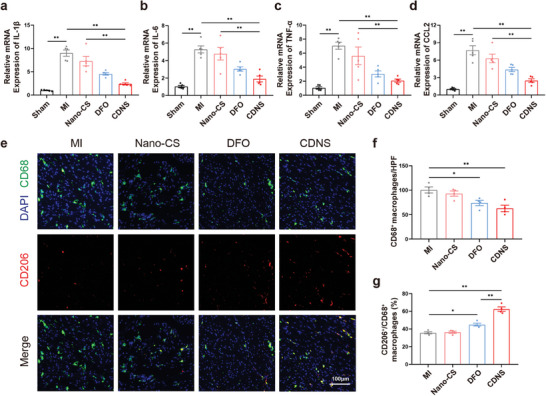
CDNS inhibited the inflammatory response of infarcted hearts after MI. a–d) Quantification of the relative mRNA expression of IL‐1β, IL‐6, TNF‐α, and CCL2 in the infarcted heart at 3 days after MI. n = 5 per group. e–g) Representative image and quantification of CD68 and CD206 immunostaining in the heart at 7 days after MI. Scale bar: 100 µm. n = 4 per group. ^*^ *p* < 0.05. ^**^ *p* < 0.01.

Excessive immune–inflammatory responses profoundly exacerbate cardiac injury and macrophage recruitment.^[^ ^27^ ^]^ Ferroptosis is implicated in intensifying inflammatory responses and macrophage infiltration.^[^ ^4b^ ^]^ Consequently, we have meticulously evaluated the inflammatory response within the myocardium post‐MI surgery. A critical surge in inflammation observed at 3 days post‐MI is a harbinger of irreversible cardiomyocyte death and detrimental structural changes, highlighting the crucial role of inflammation modulation.^[^ ^28^ ^]^ The pronounced increase in levels of the proinflammatory cytokines IL‐1β, IL‐6, TNF‐α, and CCL‐2 at this time point indicates rampant inflammation in MI patients. Remarkably, CDNS significantly decreased the levels of these cytokines compared to those in MI controls, indicating a potent attenuation of inflammation (**Figure** **6**a–d). Together with fewer proinflammatory macrophages, these findings imply that CDNS tempers inflammation, potently alleviating further damage.

Macrophages, which are essential players in MI, differentiate into proinflammatory M1 macrophages, which release cytokines early post‐MI, and into anti‐inflammatory M2 macrophages, which mediate subsequent repair.^[^ ^29^ ^]^ Total and M2 macrophages were marked with antibodies against CD68 and CD206. CDNS significantly reduced the number of CD68+ cells (Figure 6e–g). The percentage of M2 macrophages, reflected by the CD206^+^/CD68^+^ ratio, increased in the CDNS group compared with the MI control group. This result suggests that CDNS not only suppresses the proinflammatory phase of MI but also accelerates the M2‐dominated reparative transition, potentially benefiting from the properties of the chitosan hydrogel.

**Figure 7:**
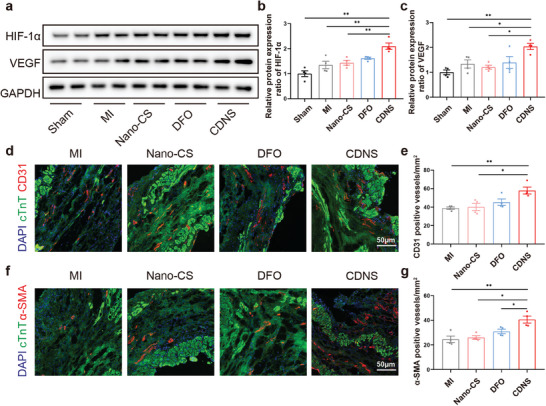
CDNS promoted angiogenesis in infarcted hearts after MI. a–c) Representative immunoblot image and quantification of the relative protein expression of HIF‐1α and VEGF in the heart at 7 days after MI. n = 4 per group. d–g) Representative image and quantification of CD31 and α‐SMA immunostaining of the heart at 28 days postoperation. Scale bar: 50 µm. n = 4 per group. ^*^ *p* < 0.05. ^**^ *p* < 0.01.

Re‐establishment of blood flow post‐MI is vital for inhibiting ventricular remodeling.^[^ ^30^ ^]^ The inhibition of ferroptosis has been shown to have a distinct proangiogenic effect.^[^ ^31^ ^]^ In this context, we assessed hypoxia‐inducible factor‐1α (HIF‐1α) and vascular endothelial growth factor (VEGF) levels on day 7 post‐MI. The expression of these genes increased significantly after treatment with CDNS (**Figure** **7**a–c), although the upregulation of HIF‐1α by DFO was not statistically significant. On day 7 post‐MI, CDNS also markedly enhanced HIF‐1α and VEGF expression compared with those in the controls. Endothelial and smooth muscle cell staining at 28 days (for CD31 and α‐SMA) ^[^ ^29^, ^32^ ^]^ revealed a greater vascular density after treatment with CDNS (Figure 7d–g). This finding suggests that CDNS extends the proangiogenic benefits of DFO, conferring more durable and effective angiogenesis during recovery. Collectively, these findings reveal the multifaceted therapeutic effects of CDNS, including antiferroptotic, anti‐apoptotic, and proangiogenic effects, and highlight its promise as a novel agent to mitigate MI damage.

### 2.8 Biocompatibility Evaluation of CDNS In Vivo

**Figure 8:**
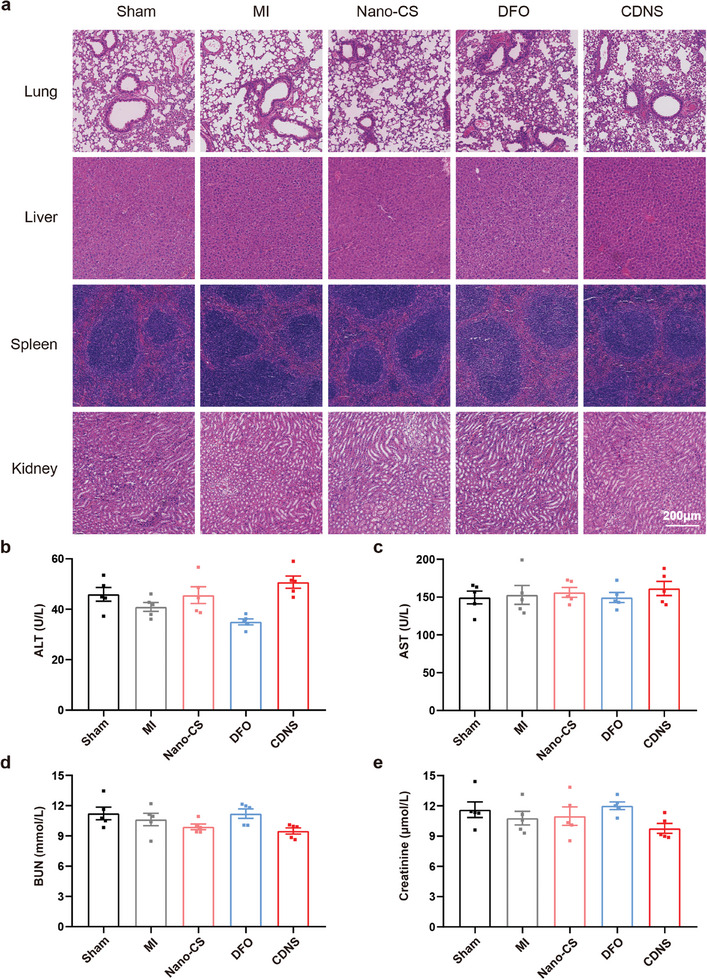
In vivo biocompatibility of CDNS in mice. a) Representative images of H&E staining of the main organs from mice treated with DMEM, Nano‐CS, DFO, and CDNS at 28 days postinjection. Scale bar: 200 µm. b–e) Serum levels of aspartate transaminase, alanine transaminase, creatinine, and blood urea nitrogen in the mice at 28 days postinjection. n = 4 per group.

We conducted in‐depth biocompatibility and safety analyses in vivo to understand the potential systemic toxicity of CDNS. Hematoxylin and eosin (H&E) staining was conducted to assess the impact of a myocardial CDNS injection on other organs. As shown in **Figure** **8**a, even at 28 days post‐CDNS treatment, myocardial injection caused no detectable inflammation, hemorrhage, or other abnormalities in the lung, liver, spleen, or kidney. In addition, we analyzed the liver function indicators of alanine transaminase (ALT) and aspartate transaminase (AST) and the kidney function indicators of urea nitrogen (BUN) and creatinine (CRE). As shown in Figure 8b–e, no significant differences in the levels of these biochemical markers were detected between treatments. Additionally, the impact of CDNS on the metabolic activity of hepatic microsomes, particularly the cytochrome P450 enzymes crucial for liver metabolism, was assessed.^[^ ^33^ ^]^ The results, as shown in Figure S11 (Supporting Information), indicate that CDNS administration did not modify the enzymatic activity of hepatic P450 enzymes, suggesting that CDNS does not disrupt hepatic metabolism. Collectively, these findings confirm that CDNS has negligible in vivo toxicity and outstanding biocompatibility for MI treatment. Critically, CDNS can be safely utilized in MI without causing systemic harm, supporting its translational potential in the clinic.

However, we must acknowledge the inherent limitations associated with the method of myocardial injection administration. While our findings do not indicate systemic toxicity, localized delivery via injection may not uniformly distribute therapeutic agents throughout the myocardium, potentially limiting the efficacy of treatment. Additionally, the invasive nature of myocardial injections could pose risks of mechanical damage or infection, albeit low, which are considerations for clinical translation. Future research could explore alternative administration routes or delivery systems that allow for more homogeneous distribution within the myocardium and reduce invasiveness to enhance the delivery and efficacy of CDNS for MI treatment.

## 3 Conclusion

We engineered a deferoxamine‐incorporated chitosan‐based nanosponge and evaluated its potential for MI treatment by targeting ferroptosis. The nanosponge directly sequestered iron regardless of DFO release because of its nanohydrogel structure composed of crosslinked CS and DFO. The iron‐chelating ability of CDNS was significantly increased compared with that of free DFO, indicating synergistic adsorption. Moreover, CDNS successfully mitigated ferroptosis and oxidative stress by efficiently chelating iron and facilitating its efflux in vitro. Our investigation indicated that CDNS administration effectively alleviated MI‐induced injury, enhanced cardiac function, and ameliorated oxidative stress‐triggered mitochondrial dysfunction, leading to a significant improvement in the survival rate of mice subjected to MI injury. Consequently, CDNS has emerged as a potential cardioprotective agent that exhibits both excellent biosafety and therapeutic potential for MI treatment through ferroptosis inhibition. In summary, our findings suggest that CDNS represents a compelling therapeutic strategy for preventing and treating MI. Given its high efficacy and good biosafety, CDNS holds great promise for future clinical applications in the management of myocardial infarction.

## 4 Experimental Section

### Preparation of CDNS

First, DFO was dissolved with high glucose Dulbecco's Modified Eagle Medium (DMEM) to make the concentration of 1 mg mL^−1^. Afterward, CS (Mn = 5000,n ≈28) was then added to the mixed solution to make the final concentration of 1.25 mg mL^−1^, following the adjustment of the pH to 7.4 with 0.1 m Tris solution. Last, the mixture was placed in the incubator for 2 h (37 °C, 5%CO_2_) to form CDNS. The obtained CDNS were collected by centrifugation at 12 000 rpm for 10 min and washed with distilled water for further tests.

### Iron Assay

To determine the iron contents of the cells or tissues, the experiment was conducted using the iron colorimetric assay kit. Briefly, the cells or tissues were first lysed by RIPA for 2 h at 4 °C. The lysates were then centrifuged at 12 000 rpm for 5 min. The supernatants were then collected and mixed with the prepared mixture provided by the kits for 1 h at 60 °C. Then the mixture was centrifuged for the collection of supernatant, which was then mixed with the detection reagent provided by the kit and incubated for 30 min at room temperature. Lastly, the supernatants of the mixture were collected and added to the 96‐well plate. The standard curve was conducted using the same procedure using the standard substance provided by the kits. The absorbance of each sample was determined by 550 nm. The iron content of each sample was calculated according to the standard curve.

### MDA Assay

The MDA assay was conducted using the commercial kit. Briefly, the cells or the tissues were first lysed by RIPA buffer and centrifuged for the collection of supernatants. Then the supernatants were mixed with the MDA working buffer and boiled at 100 °C for 15 min. After cooling to room temperature, the mixture was centrifuged again at 1000 g for 10 min, and the supernatants were collected into the 96‐well plate. Lastly, the absorbances of 532 nm of the solution were recorded. The MDA content of each sample was calculated by the standard curve and normalized by the protein concentration of the cells or the weights of the tissues.

### Endothelial Cell Tube Formation Assay

HUVECs (1 × 10^4^ cells/well) were first plated with Corning Matrigel Matrix (Corning, 356234) in 48‐well plates. After 8 h of incubation with the same volume of DMEM, Nano‐CS, DFO, and CDNS at 37 °C in a cell culture incubator, tube‐like structure formation was imaged using an inverted microscope (Nikon, Tokyo, Japan). The capillary length was quantified by measuring the length of branches from representative fields using ImageJ software.

### Performance of Echocardiography

After 7, 14, and 28 days of MI surgery, the mice were performed with echocardiography by the Vevo 1100 system (VisualSonics Inc.), which was equipped with a 30 MHz ultrasound transducer. Briefly, the mice were anesthetized by isoflurane. The cardiac function was then evaluated by calculating the ejection fraction (EF), fraction shortening (FS), left ventricular end‐systolic diameter (LVIDd), and left ventricular end‐diastolic diameter (LVIDs) under the M‐mode of the echocardiograms.

### Histological Analysis

Four weeks post‐MI surgery, the mice were sacrificed to collect the hearts. The hearts were quickly removed and washed with normal cold saline, followed by embedding in the 4% paraformaldehyde for 24 h at room temperature. Then the samples were dehydrated and embedded in the paraffin. Furthermore, the embedded samples were cut into 5 µm slices from the apex to the baseline of the hearts. The slides were then performed with hematoxylin‐eosin (H&E) staining and Masson stating to observe the morphology and collagen deposition. The fibrosis area and wall thickness were calculated with Image J. The deposition of the irons in the hearts was reflected by the Perlus blue staining.

### Immunofluorescence Staining

To examine the apoptosis cells of the heart, the TUNEL kit was applied following the manufacturer's instructions. To detect the inflammation infiltration of macrophages of the heart after 7 days of MI surgery, the slides were incubated with primary antibodies of CD206 and CD68, respectively. To verify the angiogenesis of infarct hearts after 28 days of MI surgery, the slides were incubated with the primary antibody of CD31 or α‐SMA. The primary antibody of cTnT was applied to examine the viable myocardium of the infarct heart. The slides above were stained with corresponding fluorescent dye‐conjugated secondary antibodies.

### Statistical Analysis

All experimental results were presented as the mean ± standard error of the mean (SEM) and analyzed by two‐tailed unpaired *t*‐test for comparing differences between two groups and one‐way ANOVA for comparing differences among three or more groups. *p* values < 0.05 were considered statistically significant. Statistical analysis was conducted using GraphPad Prism 8 software (GraphPad Software Inc., La Jolla, CA).

## Conflict of Interest

The authors declare no conflict of interest.

## Supporting information

Supporting Information

## Data Availability

The data that support the findings of this study are available in the supplementary material of this article.
