# Supplementary material for: Nanosponge for Iron Chelation and Efflux: A Ferroptosis‐Inhibiting Approach for Myocardial Infarction Therapy
Source: Adv Sci (Weinh). 2024 Apr 26;11(25):2305895. doi: 10.1002/advs.202305895 (PMC11220697; doi:10.1002/advs.202305895)
Supplement: Supplementary file 1 — Supporting Information [file ADVS-11-2305895-s001.pdf]

ADVANCED SCIENCE
Open Access

# Supporting Information

for *Adv. Sci.*, DOI 10.1002/advs.202305895

Nanosponge for Iron Chelation and Efflux: A Ferroptosis-Inhibiting Approach for Myocardial Infarction Therapy

*Qingbo Lv, Jun Lin, He Huang, Boxuan Ma, Wujiao Li, Jiawen Chen, Meihui Wang, Xiaoyu Wang*, Guosheng Fu* and Yun Xiao**

# Supporting Information

**Nanosponge for Iron Chelation and Efflux: A Ferroptosis-Inhibiting Approach for Myocardial Infarction Therapy**

*Qingbo Lv* †, *Jun Lin* †, *He Huang* †, *Boxuan Ma, Wujiao Li, Jiawen Chen, Meihui Wang, Xiaoyu Wang**, *Guosheng Fu**,*Yun Xiao**

† These authors contributed equally.

* To whom correspondence should be addressed.

Q. Lv, J. Lin, H. Huang, B. Ma, W. Li, J. Chen, M. Wang, G. Fu, Y. Xiao
Key Laboratory of Cardiovascular Intervention and Regenerative Medicine of Zhejiang Province
Department of Cardiology
Sir Run Run Shaw Hospital
Zhejiang University School of Medicine
Hangzhou 310016, China
E-mail: fugs@zju.edu.cn; xiaoyun@zju.edu.cn;
X. Wang
Qiushi Academy for Advanced Studies
Zhejiang University
Hangzhou 310058, China
E-mail: xy_wang@zju.edu.cn
J. Lin
Department of Cardiovascular Surgery
Sun Yat-sen Memorial Hospital
Sun Yat-sen University
Guangzhou 510120, China

**Experimental Section/Methods**

**Materials**

All reagents were used without purification. Chitosan (CS) (448869, Sigma) and deferoxamine (DFO) (D9533, Sigma) were purchased from Sigma. The DMEM was purchased from Genom Bio. The Tris HCl and ferric chloride were purchased from Macklin. The H9c2 cell lines and human umbilical vein endothelial cells (HUVECs) were purchased from ATCC. The cell counting kit-8 (CCK-8), lactate dehydrogenase (LDH) assay kit, calcein-AM/PI, and FerroOrange assay kits were purchased from Dojindo. The DHE dye was purchased from BestBio. The Annexin V-FITC/PI, JC-10 Kit, and qPCR SYBR Green Master Mix were purchased from YEASEN. The iron colorimetric assay kit was purchased from Applygen Technology. The MDA assay was purchased from Beyotime. The TUNEL kit was purchased from Roche Applied Science. Rabbit anti-GPX4 (ab125066, 1:1000 for Western blot), cTnT (ab8295, 1:200 for IF), and CD31(ab28364, 1:50 for IF) were purcahsed from Abcam. Rabbit anti-Bcl-2 (26593-1-AP, 1:1000 for Western blot), Bax (50599-2-Ig, 1:1000 for Western blot), VEGF (19003-1-AP, 1:1000 for Western blot), and Anti-GAPDH (HRP-60004, 1: 5000 for Western blot) were purchased from Proteintech. Rabbit anti-HIF-1α (371, 1:1000 for Western blot) and α-SMA (19245, 1:200 for IF) were purchased from CST. The Goat anti-CD206 (AF2535, 1:20 for IF) was purchased from R&D, and rabbit anti-CD68 (FA11, 1:200 for IF) was purchased from Bio-Rad. The CD31 (ab28364, 1:50 for IF) was purchased from Abcam. The RNA-quick purification kit was purchased from Esunbio. The PrimeScriptTM RT Master Mix was purchased from Takara. The primers were synthesized by TsingkeBiotechnology. The Matrigel Matrix for the tube formation assay was purchased from Corning.

**Characterization of CDNS**

The morphology of CDNS was observed by transmission electron microscopic (TEM) (HT-7700, Hitachi, Japan), and the size distribution of CDNS was statistically analyzed by Nano Measurer software. The size and zeta potential of CDNS were evaluated by dynamic light scattering (DLS). The transmittance and crystalline structures of CDNS were characterized by fourier transform infrared spectrometer (FT-IR) spectroscopy (Thermo, US) and X-ray diffraction (XRD) (Bruker, German), respectively. The X-ray photoelectron spectroscopy (XPS) (Shimadzu, Japan) measure was performed to identify the binding energy of corresponding peaks of CDNS. The element content of CDNS was obtained by energy dispersive spectroscopy (EDS) (S-3000 N, Hitachi, Japan).

The release characteristic of CDNS was determined by high-performance liquid chromatography (HPLC). 10 mL CDNS suspension was displaced into the Visking-MD34 dialysis bag with a rotating speed of 50 rpm/min. Then the DFO released in Tris-HCl (pH 7.4 and 6.2) was measured by HPLC at 0 h, 1 h, 2 h, 3 h, 4 h, 5 h, 6 h, 7 h, 8 h, 9 h,10 h,12 h, 14 h,16h, and18 h. The concentration of DFO in the supernatant was calculated by the standard curve.

**X-ray Photoelectron Spectroscopy (XPS) Analysis**

For the XPS analysis, CDNS were prepared at a concentration of 1.5 mg/mL. These nanoparticles were thoroughly mixed with an excess of 0.01M ferric chloride ($FeCl_3$ ) solution to ensure complete interaction. The resulting CDNS-Fe complex underwent a sequence of rigorous washes using deionized water. This stringent washing procedure was employed to eliminate any $FeCl_3$ ions that had not been chelated. The persistence of washing was continued until subsequent assays confirmed the absence of free ferric ions, thereby ensuring that the samples analyzed by XPS contained only the chelated complex.

**Determination of the Iron-Chelating Capacity of CDNS**

Initially, we measured the full UV-vis spectrum of the DFO-Fe complex (Figure S3A) and selected the maximum absorption at 430 nm as the detection wavelength. A standard curve was then generated by measuring the absorbance of the solution at 430 nm as a function of iron concentration (Figure S3B). Based on the iron detection method described above, we conducted a comparison of the iron affinity among chitosan solution, Nano-CS, DFO, and CDNS (Figure 2G).

To determine and compare the iron-chelating capacity of CDNS, Nano-CS, DFO solution, and CS solution, we established a novel detection method for iron content measurement in solutions. To be specific, 0.01M $FeCl_3$ and 0.05 mg/mL DFO solution were first mixed for 10 min at room temperature. The mixture then underwent a vis-UV spectral scan within the range of 390 nm to 700 nm by Biotek (Synergy H1) to access the Full-band spectral curve. Then different concentrations of $FeCl_3$ (0. 1μM, 0.25μM, 0.5μM, 1.0μM, 2.5μM, 5μM), prepared by diluting the stock solution with deionized water, were mixed with DFO solution using the same procedure as before, respectively. Absorbance measurements were then taken at 400 nm, 415 nm, 430 nm, 470 nm, and 490 nm, and corresponding standard curves of iron concentration versus different absorbance were established to identify the optimal detection wavelength. After that, the same volume of CDNS (CS: 1.25 ng/mL, DFO: 1 mg/mL), Nano-CS (CS: 1.25 ng/mL), DFO solution (DFO: 1 mg/mL), and CS solution (CS: 1.25 ng/mL) were mixed with iron solution (3 μM) for 10 min at room

temperature. The absorbances were measured at the optimal wavelength, and the iron-chelating capacities of these nanoparticles and solutions were calculated by the remaining free iron concentration of the mixture.

**Cell culture**

The rat embryonic ventricular myocardium-derived H9c2 cell line and HUVECs were cultured in DMEM with high glucose supplemented with 10% (v/v) FBS, 100 U/mL of penicillin, and 100 mg/mL of streptomycin at 37℃ in a humidified incubator with 95% air and 5% $CO_2$.

**Cell slicing**

For TEM analysis of the cells, H9c2 cells were fixed with 1% glutaraldehyde for 10 min, followed by being fixed with 4% glutaraldehyde for 15 min at room temperature. The cells were then fixed with 1% osmium tetroxide in 0.1 mol/L cacodylate buffer for 1 hour at room temperature, dehydrated in ethanol, and embedded in resin. After polymerization, the ultrathin sections were counterstained with uranyl acetate and lead nitrate and examined with an Inspect transmission electron microscope.

**Cell count assay CCK-8**

The H9c2 cells were first plated in the 96-well plate at the density of $10^4$/mL. The media were replaced with different levels of DFO or CDNS for 24 hours. After the treatment, the cell viability was monitored by CCK-8. Briefly, CCK-8 reagents (10 µL for each well) were added and incubated for 4 to 6 hours at 37°C. The absorbance at 450 nm was collected by a microplate reader.

**Lactate dehydrogenase assay**

The H9c2 cells were plated in the 24-well plates at the density of $2\times10^5$/mL. After the treatments, the supernatants were collected and mixed with the working buffer of the LDH assay kits for 20 min at room temperature. Then the remaining cells were lysed with 1% Triton-100 for 30 min at 37℃. The lysates were also collected and mixed with the working buffer. After the incubation, the mixtures were added with the stopping buffer. Lastly, the absorbance at 490 nm was detected by a microplate reader.

**Immunofluorescence staining**

To determine the percentage of live or dead cells, the H9c2 cells after the treatments were incubated with the calcein-AM and PI using the commercial kit. For the detection of reactive oxygen, the cells were incubated with DHE dye (BB-46052, BestBio). For the evaluation of the mitochondrial membrane potential, the JC-1 assay kit was purchased for the experiment. The cell apoptosis evaluation was conducted using the Annexin V-FITC/PI Kit.

The iron fluorescence detection was conducted using the FerroOrange Kit. The experiment was performed according to the manufacturers' instructions mentioned above. Briefly, the H9c2 cells, after the treatments, were firstly washed with PBS three times. Then the cells were incubated with the corresponding fluorochrome, which was dissolved in the DMEM. After washing for another three times, the fluorescence of the cells was detected by the fluorescence microscope or flow cytometry.

**Western blotting**

The proteins extracted from the cells and heart tissues of the mice first underwent heating denaturation with the 5× loading buffer. The protein samples were then added into the individual lanes of SDS-PAGE gels for electrophoresis. After that, the proteins separated in the SDS-PAGE gels were transferred into the 0.22 mm thick polyvinylidene fluoride (PVDF) membrane. The membrane was then immersed in the 5% nonfat milk for 1 hour at room temperature for blocking. Following that, the membrane was incubated with the corresponding primary antibody overnight at 4℃. On the second day, the membrane was incubated with the secondary antibody for 1 hour at room temperature. Lastly, the bands were detected by electro-chemiluminescence reagents using the Amersham Imager 600 system.

**Experimental animals, MI model establishment, and myocardial injection strategy**

Ten-week-old male C57 BL/6J mice were purchased from Vital River (Beijing) and housed in a standard laboratory with controlled room temperature and humidity. To establish the MI model,[18] the mice were firstly anesthetized with 0.3% pentobarbital sodium (50mg/kg) and ventilated by the rodent ventilator. The chest was opened between the third and fourth ribs to expose the heart. An 6-0 nylon suture was used to ligate the left anterior descending artery (LAD) permanently. The blanching of the heart was considered the confirmation of MI. The mice in the sham group were undergone the same procedure without ligation of LAD. Electrocardiograms (ECGs) were recorded using the Vevo 1100 system to validate the successful establishment of the MI model. After 30 min, the mice with MI surgery were then injected with 50 μl of DMEM, Nano-CS, DFO, or CDNS, respectively. The whole injected solution was separated into four sites around the border of the infarcted area. The mice were recovered in a heating pad with a constant temperature of 37℃. All animal experiments were performed following the guides of Care and Use of Laboratory Animals published by the US National Institutes of Health (NIH Publication No.85-23, revised1996) and approved by the Institutional Animal Care and Use Committee of Zhejiang University.

**Fluorescence imaging**

50 μl of free water-soluble fluorescent dye ICG or ICG labeled Nano-CS in the DMEM buffer system were ejected into the infarcted heart intramyocardially. The mice were sacrificed for monitoring the fluorescence intensity of the hearts at the 1, 3, and 7 days using *ex vivo* IVIS Spectrum System (Perkin Elmer).

**RNA extraction and reverse transcription-quantitative polymerase chain reaction (RT-qPCR)**

Total RNA was extracted from the tissue of the boarding zone of the heart post 3 days of MI surgery using the RNA-Quick Purification Kit following the manufacturer’s instructions. The purified RNA was then reverse-transcribed into cDNA by PrimeScriptTM RT Master Mix. Quantitative RT-PCR was applied for the detection of relative mRNA expression of inflammatory cytokines by using Hieff UNICON qPCR SYBR Green Master Mix on the Viia 7 system (Applied Biosystems, CA, USA). 18s expression was used for normalization. PCR products were quantified using the $2^{-\Delta\Delta CT}$ method. The primer sequences all primer sequences of target genes are listed in **Table S1**.

**Evaluation of cTnI, cTnT, CK-MB, liver and kidney function**

To investigate the effect of CDNS on the myocardial enzyme spectrum in mice post-MI, blood samples were collected from mice 24 hours after MI surgery. Additionally, to assess the impact of DMEM, Nano-CS, DFO, and CDNS on liver and kidney function, blood samples were collected after 28 days of MI surgery. The collected mouse serum was then subjected to centrifugation at 3000 rpm for 10 minutes. Subsequently, the serum was separated and collected for further analysis. The levels of cardiac troponin I (cTnI) and cardiac troponin T (cTnT) were determined using a biochemical analyzer manufactured by Siemens. Similarly, the levels of creatine kinase MB (CKMB), alanine aminotransferase (ALT), aspartate aminotransferase (AST), blood urea nitrogen (BUN), and creatinine (Cr) were measured using a biochemical analyzer (BS-120, Mindray).

**Cytochrome P450 enzymatic activity assay**

For the detection of enzymatic activity in mouse liver microsomes, we employed the Cytochrome P450 Reductase (CPR) Assay Kit following the manufacturer's instructions. The procedure involved harvesting liver tissue, wherein mice were sacrificed, and the liver tissue was swiftly excised. The excised tissue was rinsed with ice-cold CPR Assay buffer containing a protease inhibitor cocktail to prevent enzymatic degradation. Subsequently, the tissue was homogenized on ice using a homogenizer to ensure a uniform tissue suspension.Microsomal fractions were isolated by centrifugation at low and high speeds successively, removing

cellular debris and nuclei. The resulting microsomal pellet was carefully resuspended in CPR Assay buffer, yielding a concentrated microsomal suspension.

The enzymatic activity assay was then conducted by preparing a reaction mixture according to the kit instructions and adding the microsomal suspension. The reaction mixture was incubated at the specified temperature for the recommended duration. Absorbance of the reaction mixture at 460 nm was measured using a spectrophotometer at regular intervals. A standard curve, generated with known enzyme concentrations provided in the kit, was used to determine P450 enzyme activity in the microsomal fraction based on absorbance readings.

**Supplementary Tables**

**Table S1.** List of primer sequence of target gene used in RT-qPCR

| Genes | Primers（F-forward，R-Reverse） |
|---|---|
| IL-1β-Mouse | F-5'-TGAAGTTGACGGACCCCAAA-3' |
| | R-5'-TGATGTGCTGCTGTGAGATT-3' |
| IL-6-Mouse | F-5'-ACAACCACGGCCTTCCCTACTT -3' |
| | R-5'-CACGATTTCCCAGAGAACATGTG -3' |
| TNF-α-Mouse | F-5'-GGACTAGCCAGGAGGGAGAA-3' |
| | R-5'-CGCGGATCATGCTTTCTGTG-3' |
| CCL2-Mouse | F-5'-GTTAACGCCCCACTCACCTG-3' |
| | R-5'-GACCCATTCCTTCTTGGGGT-3' |
| 18s-Mouse | F-5'-AGGGTTCGATTCCGGAGAGG-3' |
| | R-5'-CAACTTTAATATACGCTATTGG-3' |

## Supplementary Figures

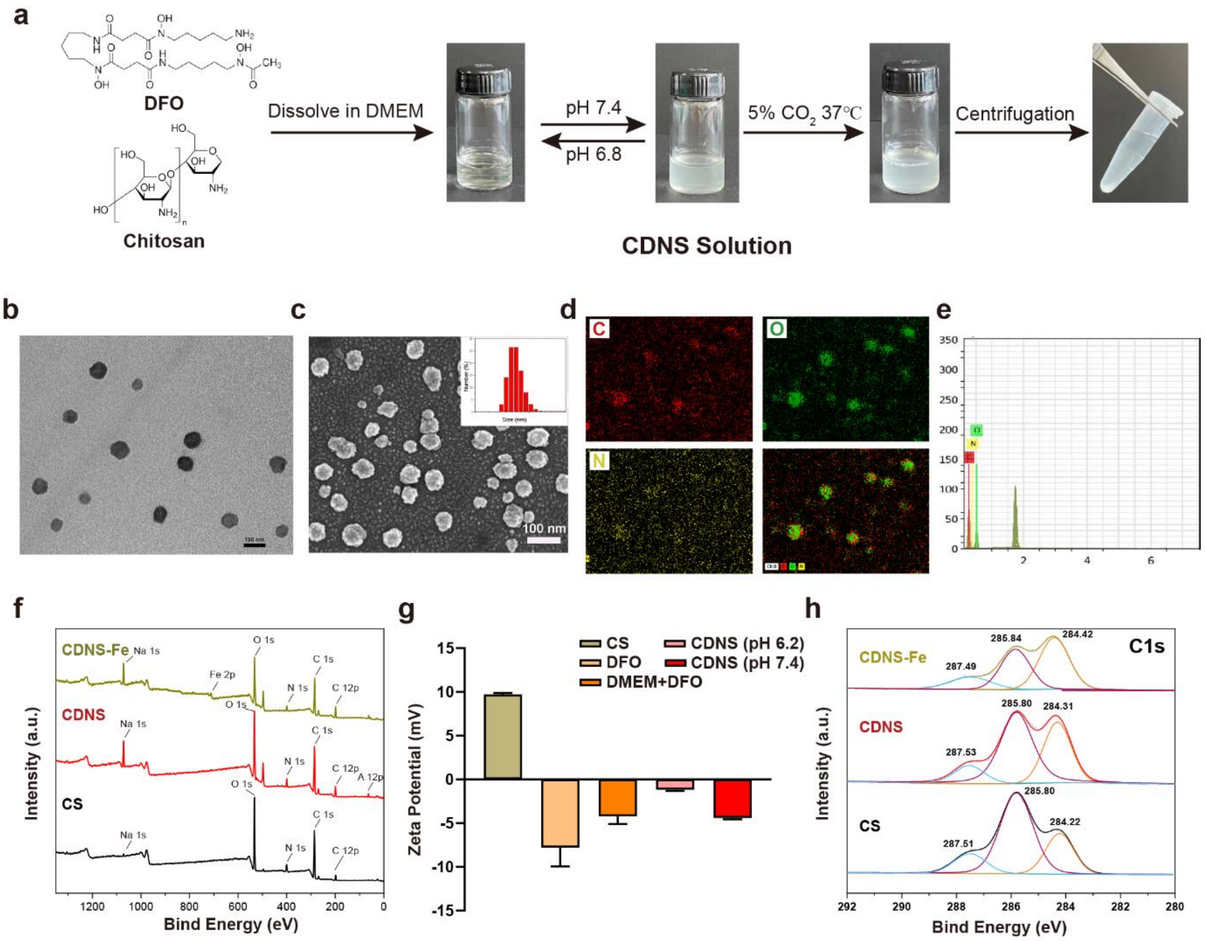


**Figure S1.** Synthesis and characterization of the CDNS. (a) The synthesis process of the CDNS and their pH stimuli responsiveness. (b) TEM image of the CDNS. Scale bar: 100 nm. (c) SEM image of the CDNS (Insert: size distribution of CDNS). Scale bar: 100 nm. (d) The elemental analysis of CDNS by energy-dispersive X-ray spectroscopy (EDS) (C: Carbon; O: Oxygen; N: Nitrogen; M: Merge). (e) EDS spectrum of CDNS. (f) X-ray photoelectron spectroscopy (XPS) analysis of all elements within CS, CDNS, and CDNS-Fe. (g) Zeta potential of CS, DFO, and CDNS at pH 6.2 and pH 7.4. (h) XPS analysis of C 1s within CS, CDNS, and CDNS-Fe.

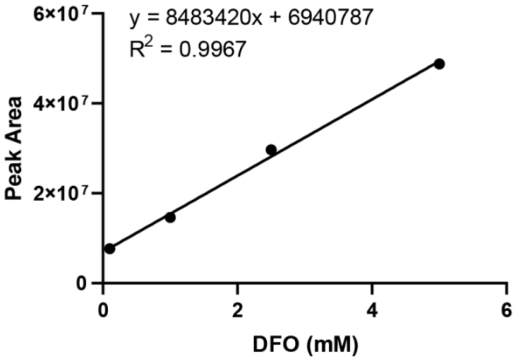


**Figure S2.** The standard curve of DFO by high-performance liquid chromatography (DFO at concentrations of 0.05, 1, 2.5, and 5 mM)

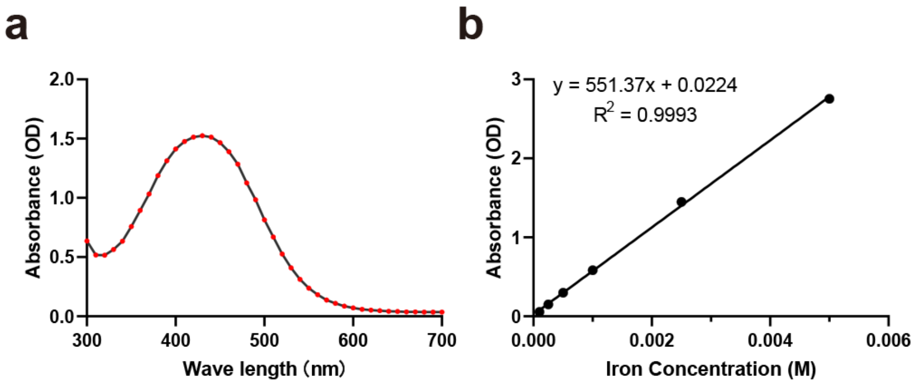


**Figure S3.** Establishment of $Fe^{3+}$ quantification. (a) UV-vis absorption spectra of Fe-DFO. (b) The standard curve of Fe-DFO by UV-vis spectrum (Fe-DFO at concentrations of 0. 25, 0. 5, 1, 2.5, and 5 mM) at 430 nm.

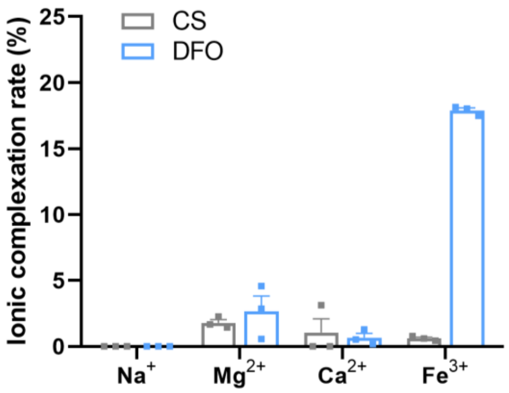


**Figure S4.** Different metallic ions ($Na^{+}$, $Mg^{2+}$, $Ca^{2+}$, $Fe^{3+}$) chelation efficiency of CS and DFO. n=3 per group.

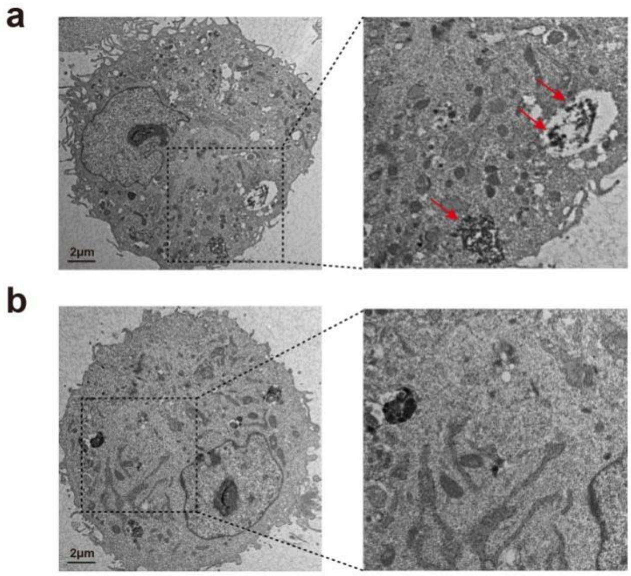


**Figure S5.** Representative TEM images of H9c2 cells treated with CDNS or DFO for 30 min. (a) Representative TEM images of H9c2 cells treated with CDNS for 30 min. The red arrows indicate CDNS. (b) Representative TEM images of H9c2 cells treated with DFO for 30 min. Scale bar: 2 μm.

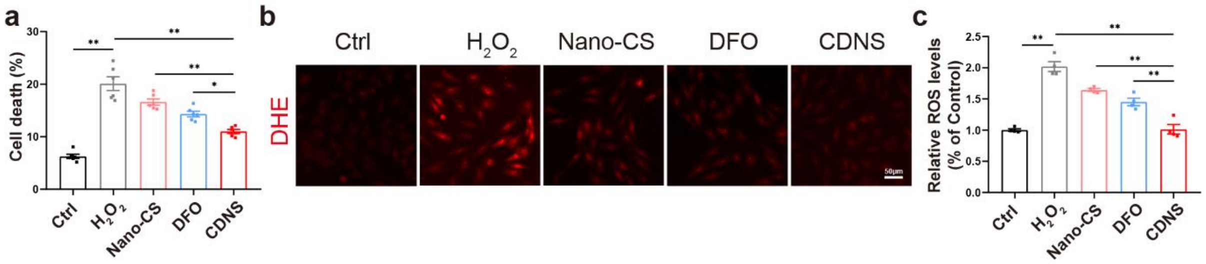


**Figure S6.** CDNS protected H9c2 from oxidative damage. (a) Relative viability of H9c2 cells pretreated with DMEM, Nano-CS, DFO, and CDNS for 2 hours and then treated with 200 μmM $H_2O_2$ for 24 h. n=6 per group. (b-c) Immunofluorescence analyses evaluate the reactive oxygen species (ROS) of H9c2 cells after different treatments by DHE staining. Scale bar: 50 μm. n=4 per group. *$P$ < 0.05. **$P$ < 0.01.

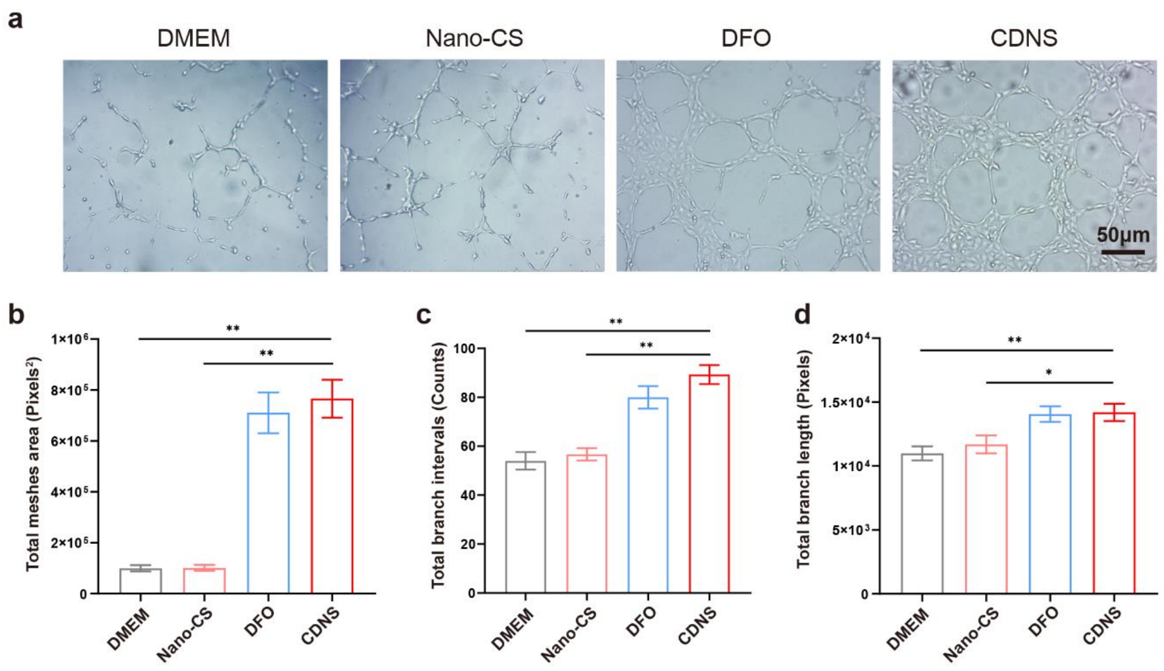


**Figure S7.** *In vitro* HUVECs tube-like formation assay. (a) The microscopic images of HUVECs tubular structure formation. Scale bar: 50 μm. (b-d) The quantified result of total meshes area, total branch intervals, and total branch length. n=4 per group. **P* < 0.05. ***P* < 0.01.

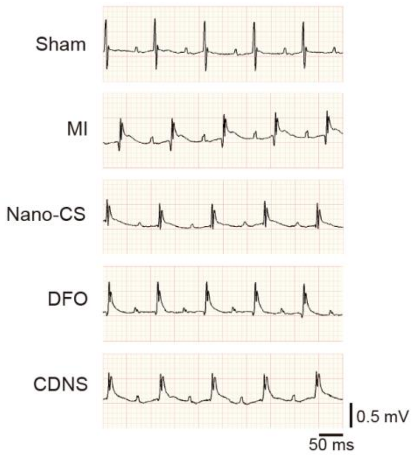


**Figure S8.** The electrocardiograph (ECG) of mice from different groups after Sham or MI surgery. The typical ECG features of MI characterized by widened QRS complex and decreased R-S segment's amplitude.

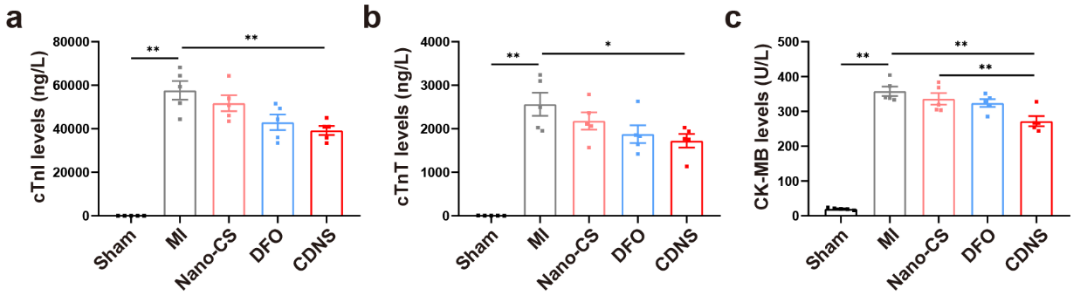


**Figure S9.** CDNS reduced the myocardial enzyme profiles after MI surgery. (a-c) The levels of cTnI, cTnT, and CK-MB in serum of mice were examined 24 hours post-MI surgery. n=5 per group. **P* < 0.05. ***P* < 0.01.

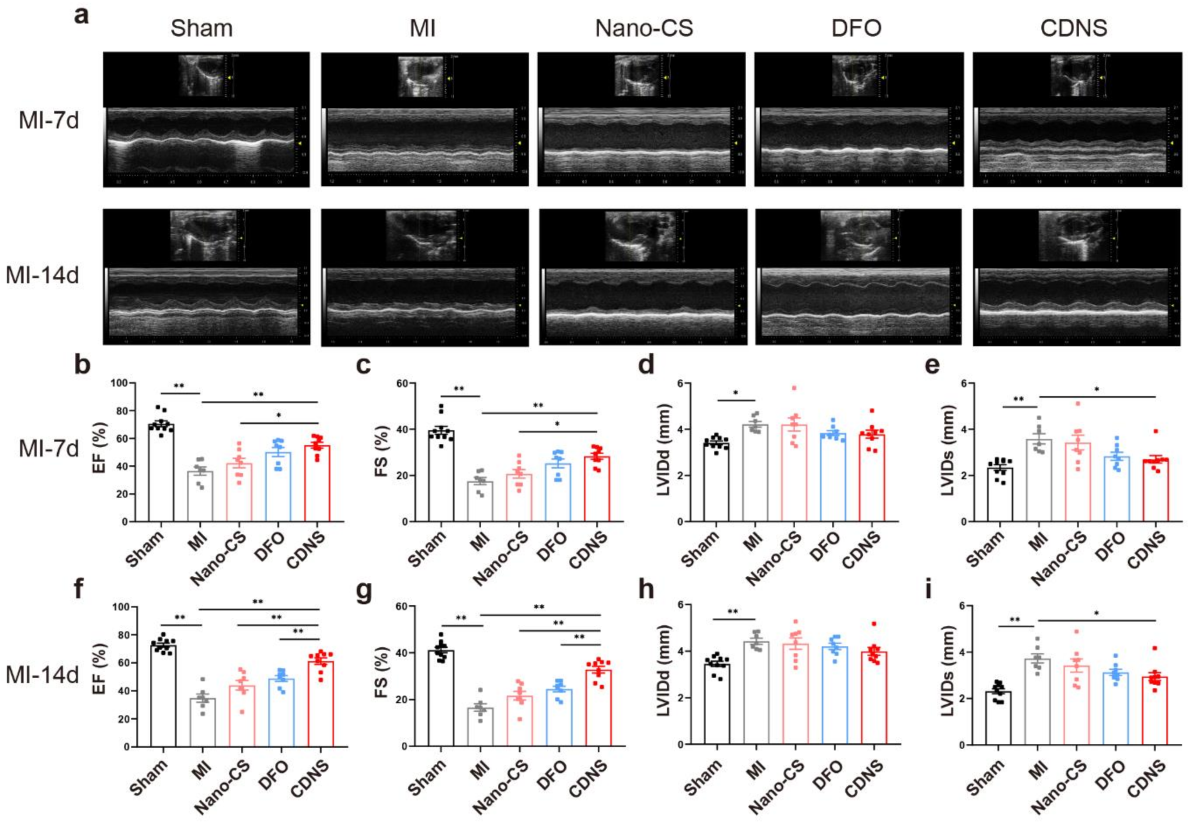


**Figure S10.** CDNS reserves cardiac function and inhibits the infarcted area of the hearts after MI. (a) Representative echocardiography images of the hearts in different groups at 7 and 14 days after MI. (b-i) Quantification analysis of EF, FS, LVIDd, and LVIDs of the hearts in different groups at 7 and 14 days after MI, respectively. n=10 for sham group, n=7 for MI group, n=8 for Nano-CS group, n=8 for DFO, n=9 for CDNS group. *$P < 0.05$. **$P < 0.01$.

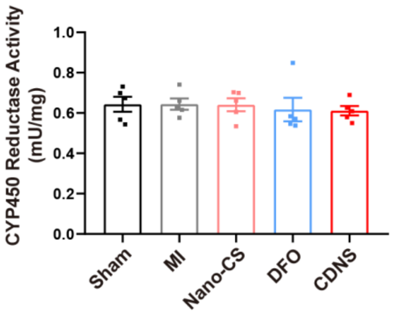


**Figure S11.** The liver CYP450 reductase activity of mice from different treatment groups. n=5 per group.
